# Loss of the N-terminal methyltransferase NRMT1 increases sensitivity to DNA damage and promotes mammary oncogenesis

**DOI:** 10.18632/oncotarget.3653

**Published:** 2015-03-26

**Authors:** Lindsay A. Bonsignore, Jill Sergesketter Butler, Carolyn M. Klinge, Christine E. Schaner Tooley

**Affiliations:** ^1^ Department of Biochemistry & Molecular Genetics, Center for Genetics and Molecular Medicine, University of Louisville School of Medicine, Louisville, KY, USA

**Keywords:** DNA damage, DNA repair, breast cancer, N-terminal methylation, NRMT1

## Abstract

Though discovered over four decades ago, the function of N-terminal methylation has mostly remained a mystery. Our discovery of the first mammalian N-terminal methyltransferase, NRMT1, has led to the discovery of many new functions for N-terminal methylation, including regulation of DNA/protein interactions, accurate mitotic division, and nucleotide excision repair (NER). Here we test whether NRMT1 is also important for DNA double-strand break (DSB) repair, and given its previously known roles in cell cycle regulation and the DNA damage response, assay if NRMT1 is acting as a tumor suppressor. We find that NRMT1 knockdown significantly enhances the sensitivity of breast cancer cell lines to both etoposide treatment and γ-irradiation, as well as, increases proliferation rate, invasive potential, anchorage-independent growth, xenograft tumor size, and tamoxifen sensitivity. Interestingly, this positions NRMT1 as a tumor suppressor protein involved in multiple DNA repair pathways, and indicates, similar to BRCA1 and BRCA2, its loss may result in tumors with enhanced sensitivity to diverse DNA damaging chemotherapeutics.

## INTRODUCTION

We have recently discovered the first eukaryotic N-terminal methyltransferase, NRMT1 [1]. NRMT1 is a highly conserved, nuclear trimethylase expressed in all tissues [2]. After cleavage of the initiating methionine, it methylates the α-amino group of the newly exposed N-terminal amino acid [1]. NRMT1 was originally found to methylate an N-terminal X-Pro-Lys consensus sequence, with X being any amino acid besides Leu, Ile, Trp, Asp, or Glu [1]. We have subsequently verified an extended NRMT1 consensus sequence, which accepts most uncharged polar or nonpolar amino acids at the second position and either Lys or Arg in the third position [3]. This extended consensus results in over 300 predicted NRMT1 targets, including genes involved in chromatin structure (CENPA, CENPB, HP1γ, SET) and DNA repair (Rb, DDB2, PARP3, BAP1).

Upon the initial discovery of N-terminal methylation almost forty years ago [4, 5], it was primarily thought to protect proteins against cellular proteases and to serve as a general mediator of protein stability [6, 7]. However, in identifying NRMT1, we have been able to demonstrate that N-terminal methylation can also regulate protein-DNA interactions [1, 8]. We found that loss of N-terminal methylation of regulator of chromosome condensation 1 (RCC1) reduced its affinity for DNA and resulted in multi-polar spindle formation, aberrant mitotic division, and aneuploidy [1, 8]. Subsequently, it has been shown that N-terminal methylation of Centromere protein B (CENP-B) regulates its binding to centromeric DNA and is enriched in response to cellular stress [9], and N-terminal methylation of DNA damage-binding protein 2 (DDB2) is necessary for its recruitment to foci of UV-induced DNA damage and promotes efficient nucleotide excision repair (NER) [10]. Given that N-terminal methylation produces a positive N-terminal charge, independent of local pH, and is found at points of interaction between proteins in large multi-subunit complexes, it is also proposed to regulate electrostatic protein/protein interactions [6].

The role of N-terminal methylation in DNA repair is not surprising given the accumulating evidence for the involvement of other types of protein methylation [11]. It has been shown in yeast, that methylation of histone H3 lysine 79 (K79) by Dot1 is needed for efficient repair of UV lesions [12]. In mammals, methylation of histone H3 lysine 36 (K36) by SETD2 is necessary for recruitment of C-terminal binding protein interacting protein (CtIP) to double strand breaks (DSBs), DSB resection, and efficient homologous recombination (HR) [13]. Non-homologous end joining (NHEJ) is regulated by histone H4 lysine 20 (K20) dimethylation by PR-Set7 and Suv4-20, which recruits 53BP1 and promotes NHEJ over HR [14]. Methylation of Retinoblastoma protein (Rb) on lysine 810 also recruits 53BP1, and is necessary for the participation of Rb in DNA damage response [15]. Histone H3 lysine 4 (K4) methylation recruits the tumor suppressor ING to double strand breaks [16], and the histone H3 lysine 27 (K27) methyltransferase EZH2 appears necessary for inhibiting transcription at sites of damage [17].

As DNA damage leads to accumulation of mutations and genomic instability, many proteins that promote efficient DNA repair are also important tumor suppressors. In addition to the recruitment of 53BP1 to sites of DSBs by methylated Rb [15], p53 is directly involved in NER-mediated removal of UVC-induced DNA adducts through its binding of Xeroderma Pigmentosum B (XPB) [18]. p53 also directly binds DNA polymerase β (Polβ) and facilitates its loading onto abasic DNA during base excision repair (BER), and appears to control the fidelity of both HR and NHEJ [19, 20]. Adenomatous polyposis coli (APC) also regulates BER through an interaction with Polβ and flap endocnuclease 1 (Fen-1) [21]. Phosphatase and tensin homolog deleted on chromosome 10 (PTEN) binds and regulates the sumoylation of Rad52 during DSB repair [22]. The E3-ubiquitin ligase BRCA1 is necessary for repair of DSBs through both the HR and NHEJ pathways [23, 24], and BRCA2 facilitates the nucleation of Rad51 at sites of DSBs [25]. Though loss of these tumor suppressors results in malignant growth [26], they can also provide some therapeutic benefit by enhancing sensitivity to DNA damaging chemotherapeutics [27].

As NRMT1 has already been shown to regulate NER through its methylation of DDB2 [10], and two more of its substrates (BAP1 and PARP3) function in HR and NHEJ, respectively, the goal of this study was to determine if NRMT1 is a tumor suppressor involved in multiple modes of DNA repair. We observe that loss of N-terminal methylation enhances breast cancer cell sensitivity to DSBs induced by etoposide and γ-irradiation and results in the persistence of γH2AX foci after etoposide treatment. We also observed that NRMT1 loss in these cells increases proliferation, invasive potential, anchorage-independent growth, xenograft tumor size, and sensitivity to tamoxifen. These studies indicate that NRMT1 is a tumor suppressor that is needed for both NER [10] and DNA double strand break repair. They also show that loss of NRMT1 has the potential to be a biomarker for patients more likely to respond to DNA damaging agents and less likely to acquire resistance to tamoxifen.

## RESULTS

### Loss of NRMT1 promotes sensitivity to double-strand DNA breaks

**Figure 1:**
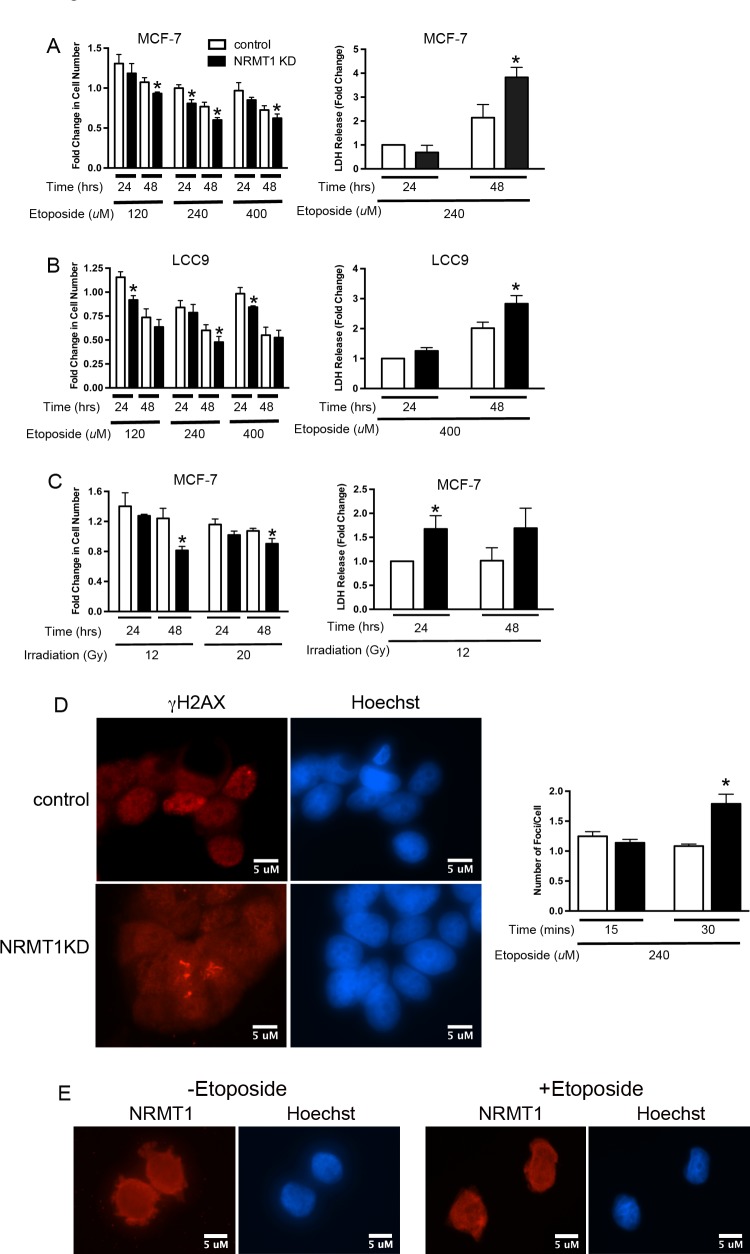
NRMT1 loss promotes sensitivity to double-strand DNA breaks (**A**) Fold change in cell number of MCF-7 NRMT1 KD (black bars) and control (white bars) cells after treatment with 120, 240, and 400 μM etoposide and corresponding LDH release of MCF-7 NRMT1 KD and control cells after treatment with 240 μM etoposide. Fold change in LDH release was calculated by setting the control at 24 hours equal to one. (**B**) Fold change in cell number of LCC9 NRMT1 KD (black bars) and control (white bars) cells after treatment with 120, 240, and 400 μM etoposide and corresponding LDH release of LCC9 NRMT1 KD and control cells after treatment with 400 μM etoposide. Fold change in LDH release was calculated by setting the control at 24 hours equal to one. (**C**) Fold change in cell number of MCF-7 NRMT1 KD and control cells after treatment with 12 and 20 Gy γ-irradiation and corresponding LDH release of MCF-7 NRMT1 KD and control cells after treatment with 12 Gy γ-irradiation. Fold change in cell number was calculated by normalizing to transduced MCF-7 cells with no treatment. Fold change in LDH release was calculated by setting the control at 24 hours equal to one. Each bar represents the mean ± SEM of three to four independent experiments. Statistical analysis was by Student's t-test, * denotes p < 0.05. (**D**) Representative image of immunofluorescence showing more γH2AX foci persist in NRMT1 KD cells 30 min after etoposide treatment as compared to control cells. γH2AX staining is shown in red, Hoechst counterstaining is shown in blue. Cells with foci were counted, and the number of foci per cell calculated 15 and 30 min post-treatment with 240 μM etoposide. Statistical analysis was by Student's t-test, * denotes p < 0.05. (**E**) NRMT1 localization does not change after 240 μM etoposide treatment, and no NRMT1 foci are observed. NRMT1 immunostaining is shown in red, Hoechst counterstaining is shown in blue.

**Figure 2:**
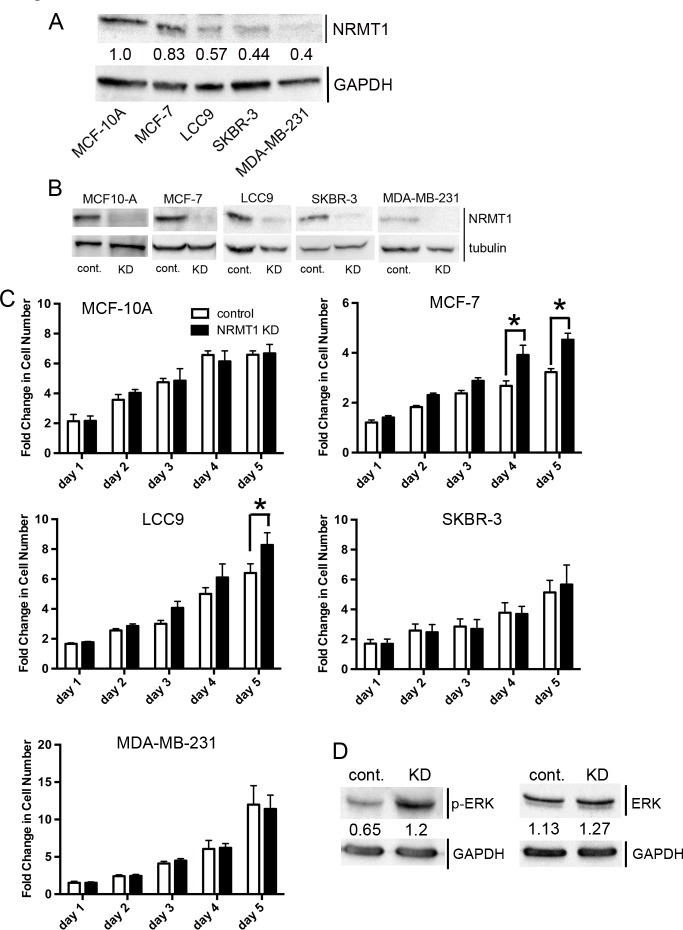
NRMT1 knockdown promotes growth of ER positive breast cancer cell lines (**A**) Protein expression levels of NRMT1 and GAPDH (loading control) of all cell lines studied. Ratio of NRMT1 levels compared to GAPDH shown as numbers below each NRMT1 band. (**B**) Protein expression confirming knockdown of NRMT1 in MCF-10A, MCF-7, LCC9, SKBR-3, and MDA-MB-231 cells treated with lentivirus expressing an shRNAmir against NRMT1 compared to cells treated with control lentivirus. α-tubulin was used as a loading control. (**C**) Fold change in cell number of MCF-10A, MCF-7, LCC9, SKBR-3, and MDA-MB231 cells treated with lentivirus expressing an shRNAmir against NRMT1 (black bars) compared to the same cell lines treated with control lentivirus (white bars). Fold change was calculated by dividing by the measurements at day zero. Each data point represents the mean ± SEM of three independent experiments. Statistical analysis was by Two-Way Anova, * denotes p < 0.05. (**D**) Western blot showing levels of phosphorylated ERK (p-ERK) increase with NRMT1 KD, though total ERK levels remain the same. GAPDH is used as a loading control. Ratio of p-ERK or total ERK levels compared to GAPDH are shown as numbers below each ERK band.

N-terminal methylation of DDB2 by NRMT1 is necessary for its recruitment to UV-induced DNA damage and proper execution of NER [10]. Additional NRMT1 targets, BRCA1 associated protein 1 (BAP1) and poly-ADP-ribosylase 3 (PARP3), are involved in DNA double strand break repair. BAP1 is a deubiquitinating enzyme recruited to DNA and required for appropriate assembly of homologous recombination factors during DSB [28]. PARP3 poly-ADP-ribosylates proteins at DSBs and promotes NHEJ [29, 30]. Therefore, we wanted to test the hypothesis that N-terminal methylation by NRMT1 is also needed for repair of DNA double strand breaks. To test this, we stably knocked down NRMT1 in MCF-7 or LCC9 human breast adenocarcinoma cells and assayed their sensitivity to etoposide treatment or γ-irradiation. The cells were transduced with either a lentivirus expressing an shRNAmir against human NRMT1 or a control lentivirus expressing an shRNAmir against mouse NRMT1 (Figure 2B). This control hairpin has previously been shown ineffective against human NRMT1 [1]. Transduced cells were treated with either etoposide (120, 240, or 400 μM) or γ-irradiation (12 or 20 Gy) and fold change in cell number from day 0 was measured 24 and 48 hours post-treatment. We found that NRMT1 knockdown (KD) cells (both MCF-7 and LCC9) were more sensitive to etoposide and showed a significant decrease in cell number at all three etoposide concentrations (Figure 1A and 1B). To monitor if this decrease in cell number corresponded with an increase in cell death and not simply slowed proliferation, we measured the release of lactate dehydrogenase (LDH) after etoposide treatment. Etoposide significantly increased LDH release from both NRMT1 KD lines at 48 hours (Figure 1A and 1B).

Similar to etoposide treatment, NRMT1 KD cells were more sensitive to γ-irradiation and showed a significant decrease in cell number 48 hours post-treatment as compared to control cells (Figure 1C). This was true at both 12 and 20 Gy, and also corresponded to a significant increase in LDH release (Figure 1C). These data suggest that NRMT1 loss sensitizes cancer cells to agents that produce DSBs. To verify that loss of NRMT1 is impeding repair of DSBs, we assayed whether γH2AX foci (markers of DSBs) persist longer after etoposide treatment in NRMT1 KD cells. We found that in the cells with visible damage, NRMT1 KD resulted in a significantly higher number of γH2AX foci per cell 30 minutes after etoposide treatment as compared to control cells (Figure 1D), indicating these foci were beginning to accumulate and DSB repair is impaired. To better understand how NRMT1 is promoting recruitment of its substrates to sites of DNA damage, we assayed whether NRMT1 itself is recruited to γH2AX foci in response to etoposide treatment. However, we found no difference in NRMT1 localization with or without etoposide treatment and were not able to detect NRMT1-containing foci (Figure 1E). Based on these data, we hypothesize that NRMT1 is not recruiting its substrates to sites of DNA damage through direct binding, but rather, the actual N-terminal methylation of substrates attracts them to DNA. Whether this is by mediating direct binding to DNA or mediating new protein/protein interactions remains to be tested.

### Knockdown of NRMT1 promotes growth of ER+ breast cancer cell lines

While loss of genes involved in DNA repair can render cancer cells more susceptible to DNA-damaging chemotherapies, many are also tumor suppressors because their loss results in an increase in genomic instability and oncogenic growth. Therefore, we tested the hypothesis that NRMT1 is acting as a tumor suppressor, and its loss will promote malignant phenotypes, including increased growth rates, invasive potential, and anchorage-independent growth. We performed these experiments in immortalized MCF10A human mammary epithelial cells and a panel of human breast cancer cell lines, including MCF-7, LCC9, SKBR-3, and MDA-MB-231. These different cell lines were chosen because they represent a sampling of different breast cancer types, and it remained unclear if NRMT1 loss alone is tumorigenic or if it worked synergistically with other mutations. MCF-7 and LCC9 human adenocarcinoma cell lines are estrogen receptor (ER) and progesterone receptor (PR) positive but do not overexpress human epidermal growth factor receptor 2 (HER2) [31]. Additionally, MCF-7 cells are sensitive to endocrine therapy, while LCC9 cells are endocrine resistant [31]. SKBR-3 cells overexpress HER2 and do not express ER or PR. MDA-MB-231 cells are triple negative (ER-, PR-, HER2) and have completed epithelial to mesenchymal transition (EMT) [32, 33]. Basal NRMT1 levels were measured in each cell line by western blot analysis (Figure 2A). Interestingly, the MCF-10A cells have the highest NRMT1 protein levels, and these levels steadily decreased as the cell lines became more aggressive in phenotype (Figure 2A). This is consistent with our hypothesis that loss of NRMT1 promotes oncogenic behavior in mammary epithelial cells.

Next, we stably knocked down NRMT1 expression in MCF-10A cells (Figure 2B) and compared cell growth between the NRMT1 KD MCF-10A cells and MCF-10A cells treated with control lentivirus. We found no differences between the two lines, indicating NRMT1 loss alone does not promote growth in non-transformed mammary epithelial cells (Figure 2C). We then similarly knocked down NRMT1 in MCF-7, LCC9, SKBR-3, and MDA-MB-231 cells (Figure 2B). While, NRMT1 KD significantly increased growth after 4-5 days in MCF-7 and LCC9 cells, it had no effect on the normally low NRMT1-expressing SKBR-3 and MDA-MB-231 cells (Figure 2C). These data suggest that loss of NRMT1 is able to promote growth in already transformed ER+ and PR+ cells lines that normally express NRMT1 at levels comparable to non-transformed mammary cells. As the proven NRMT1 target SET [1] is a known inhibitor of PP2A [34], which in turn results in increased MAP kinase signaling [35], we tested if one factor contributing to the increased cell growth was abnormal activation of MAP kinase proteins in NRMT1 KD cells. Indeed, we found that phospho-ERK levels are increased upon NRMT1 KD in MCF-7 cells (Figure 2D), indicating abnormal N-terminal methylation of SET may be contributing to this phenotype.

**Figure 3:**
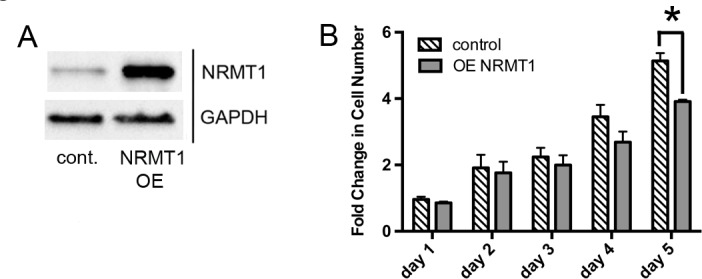
NRMT1 overexpression decreases growth of MDA-MB-231 cells (**A**) Protein expression confirming NRMT1 overexpression (OE) in MDA-MB-231 cells treated with lentivirus overexpressing NRMT1 compared to cells treated with control lentivirus. GAPDH is used as a loading control. (**B**) Fold change in cell number of MDA-MB231 cells over-expressing NRMT1 compared to cells treated with control lentivirus. Fold change was calculated by dividing by the measurements at day zero. Each data point represents the mean ± SEM of three independent experiments. Statistical analysis was by Two-Way Anova, * denotes p < 0.05.

To test if NRMT1 re-expression into cells with low NRMT1 expression can slow their growth, we transduced MDA-MB-231 cells (which have a basally high growth rate) with a lentiviral construct that overexpresses NRMT1 (Figure 3A). We then compared cell growth between the NRMT1 overexpressing MDA-MB-231 cells and cells treated with lentivirus overexpressing empty vector. Overexpression of NRMT1 significantly reduced MDA-MD-231 cell growth at 5 days (Figure 3B). This data confirms that expression of NRMT1 has at least one characteristic of a tumor suppressor. Its expression normally keeps cell growth rates in check, and its loss results in increased proliferation.

**Figure 4:**
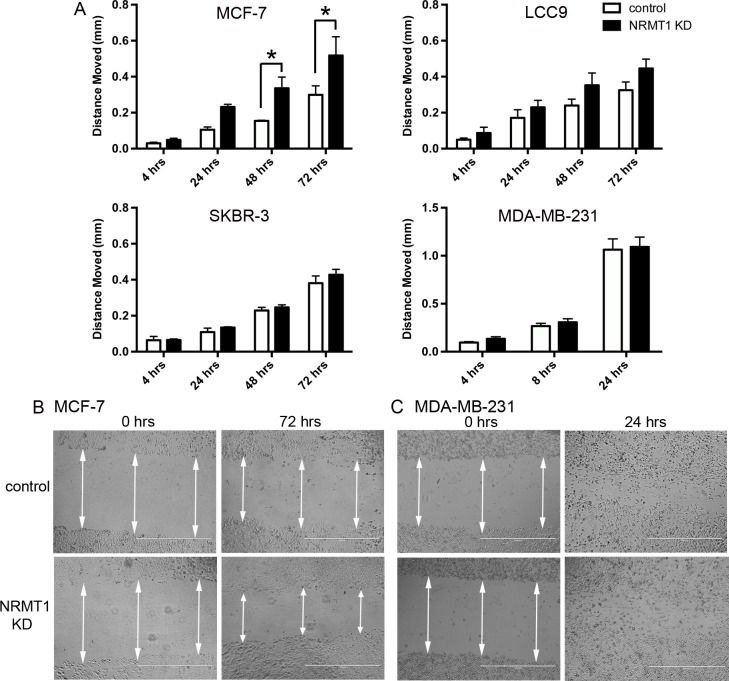
Knockdown of NRMT1 in ER positive breast cancer cell lines also increases wound filling capacity (**A**) Distance moved in the scratch-wound migration assay of NRMT1 KD MCF-7, LCC9, SKBR-3, and MDA-MB-231 cells (black bars) versus control cells (white bars). Distanced moved was calculated by subtracting scrape widths at the indicated time points from the initial scrape width. (**B**) Representative phase contrast images of MCF-7 NRMT1 KD cells and MCF-7 control treated cells at 0 hours and 72 hours in the scratch wound migration assay. (**C**) Representative images of MDA-MB-231 NRMT1 KD cells and MDA-MB-231 control treated cells at 0 hours and 24 hours in the scratch wound migration assay. White arrows denote scrape width and indicate where triplicate measurements were taken. Each data point represents the mean ± SEM of three independent experiments. Statistical analysis was by Two-Way Anova, * denotes p < 0.05.

To next test whether knockdown of NRMT1 could increase breast cancer cell migration, we performed scratch-wound migration assays. MCF-7, LCC9, SKBR-3, and MDA-MB-231 cells were again infected with NRMT1 KD or control lentivirus and assayed for their ability to fill a scratch wound. As in the viability assays, the MCF-7 cells more quickly filled the wound when depleted for NRMT1 expression (Figure 4A and 4B). The LCC9 cells showed a similar trend, though this change was not statistically significant (Figure 4A). Neither the SKBR-3 nor the MDA-MB-231 cells had a detectable difference in wound filling between the NRMT1 KD and control lentivirus transduced lines (Figure 4A and 4C). These data suggest that NRMT1 loss in breast cancer cells that still express ER and PR can promote cell migration. However, the results from the scratch-wound assay could also reflect increased cell proliferation, so we additionally measured the effect of NRMT1 KD on cell invasion.

### Knockdown of NRMT1 promotes invasive potential and anchorage independent growth of ER- breast cancer cells

**Figure 5:**
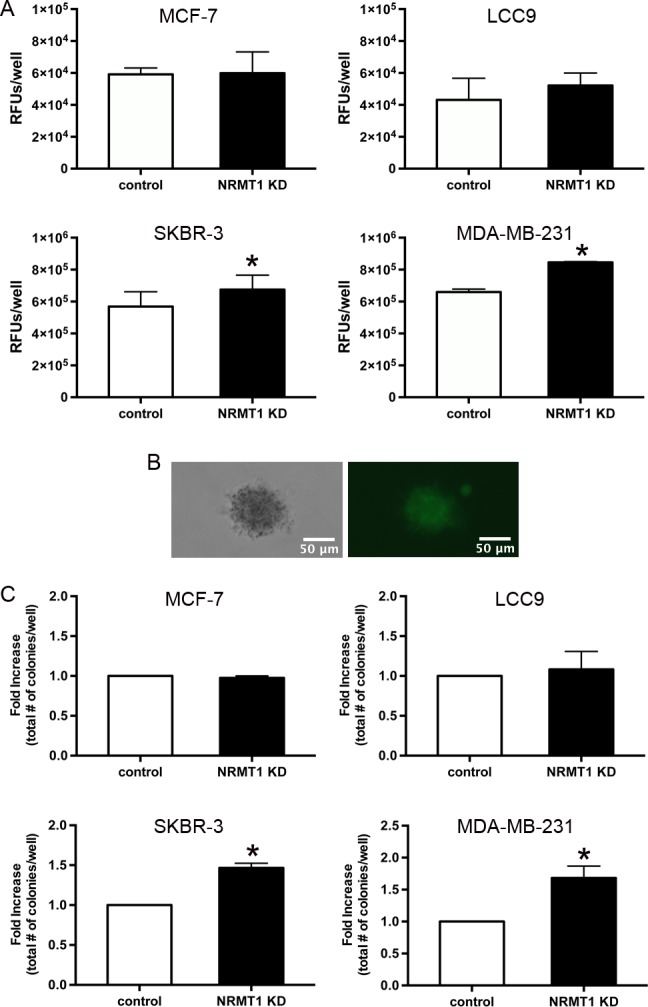
Knockdown of NRMT1 promotes invasive potential and anchorage independent growth of ER negative breast cancer cells (**A**) Invasion potential of NRMT1 knockdown MCF-7, LCC9, SKBR-3, and MDA-MB-231 cells (black bars) versus control cells (white bars) 48 hours after addition to chamber. RFU denotes relative fluorescent units. (**B**) Phase contrast and GFP fluorescence images of an MDA-MB-231 NRMT1 KD colony that is GFP positive and has a diameter greater than 50 μm. (**C**) Colony formation in a soft agarose gel of NRMT1 knockdown MCF-7, LCC9, SKBR-3, and MDA-MB-231 cells (black bars) versus control cells (white bars) at 4 weeks (all cell lines except MCF-7) or 5 weeks (MCF-7). Fold increase in the total number of colonies per well was calculated by setting control values equal to one for each cell line. Each data point represents the mean ± SEM of three independent experiments. Statistical analysis was by Student's t-test, * denotes p < 0.05.

To confirm that NRMT1 depletion promotes breast cancer migration, we tested all cell lines treated with NRMT1 KD or control lentivirus for their ability to invade a basement membrane. In contrast to the results observed above, NRMT1 KD had no effect on the invasion of MCF-7 and LCC9 cells (Figure 5A). Conversely, NRMT1 KD significantly increased the invasion of SKBR-3 and MDA-MB-231 cells (Figure 5A). These results indicate that the stimulatory effect of NRMT1 loss on cell mobility in the scratch wound assay for MCF-7 and LCC9 cells resulted from increased proliferation and not increased migratory potential. However, these results also suggest that NRMT1 depletion in the more oncogenic, ER- breast cancer cell lines results in an increased metastatic potential.

Another hallmark of advanced oncogenesis is the development of anchorage-independent growth. We transduced the four breast cancer cell lines with NRMT1 KD and control lentivirus and assayed their ability to form colonies in soft agarose. As with the cell invasion assays, NRMT1 depletion had no effect on the colony forming abilities of MCF-7 or LCC9 cells (Figure 5C). However, NRMT1 depletion significantly increased the number of colonies formed by both SKBR-3 and MDA-MB-231 cells (Figure 5B and 5C). These data support a model whereby lowered NRMT1 expression in ER+, less aggressive breast cancer cells promotes increased cell growth. In more oncogenic, ER- cell lines, NRMT1 loss promotes increased metastatic potential and anchorage independent growth. Taken together, these results suggest that NRMT1 is a tumor suppressor and that the time point at which its expression becomes altered and the type of cancer cell in which it occurs are important factors in the resultant phenotypes.

### NRMT1 depletion promotes tumor growth *in vivo*

**Figure 6:**
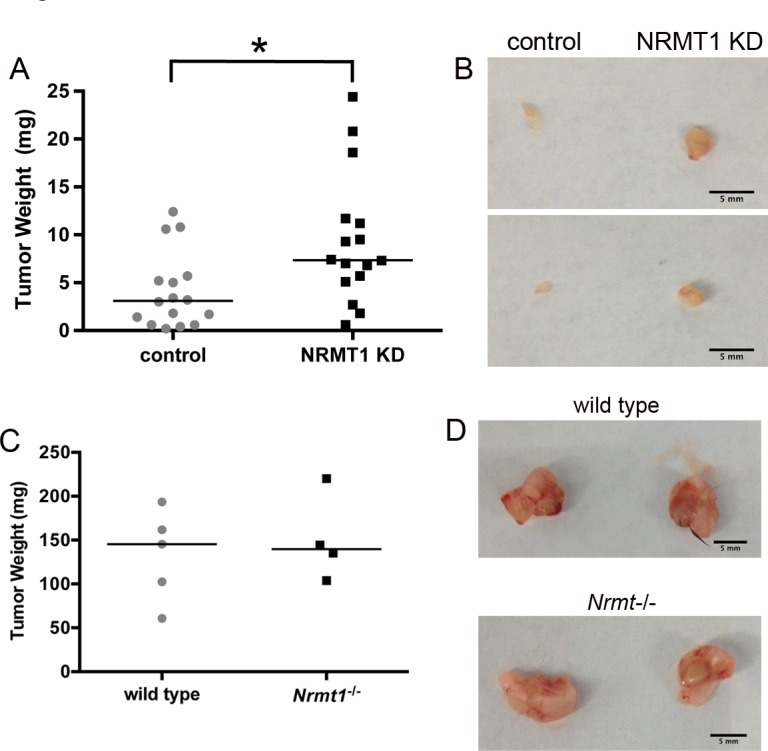
Cell autonomous NRMT1 depletion increases tumor growth *in vivo* (**A**) Tumor weights 1 week after implantation of MCF-7 NRMT1 KD or MCF-7 control cells into the mammary fat pads of Nu/J immunocompromised nude mice. NRMT1 KD cells were injected into the left inguinal gland, while control cells were injected into the right inguinal gland of the same mouse. Each symbol represents one mouse and horizontal lines denote median values. Statistical analysis was by Student's t-test, * denotes p < 0.05. (**B**) Representative images of tumors formed on each side of the same mouse 1 week after implantation. (**C**) Tumor weights 1 week after implantation of LLC1 cells into the mammary fat pads of wild type and *Nrmt1*^−/−^ C57BL/6 mice. Each symbol represents one mouse and horizontal lines denote median values. No difference in tumor weight was observed. (**D**) Representative images of tumors formed in the wild type and *Nrmt1*^−/−^ mice.

To determine if the increase in oncogenic phenotypes observed upon NRMT1 loss in cell culture leads to increased tumor size *in vivo*, we used a mouse xenograft model where MCF-7 cells were injected into the mammary fat pads of Nu/J nude mice. MCF-7 cells were selected, as they had the most pronounced increase in cell growth upon NRMT1 knockdown. MCF-7 cells were treated with the control and NRMT1 KD lentivirus as described above. For each mouse, control cells were implanted into the right mammary fad pad and experimental cells into the left. This allowed us to compare tumors grown in the same mouse for the same amount of time. We observed that MCF-7 NRMT1 KD cells formed significantly larger tumors compared to control cells after only one week of *in vivo* growth (Figure 6A and 6B), confirming that cell autonomous NRMT1 loss can promote increased tumor size *in vivo*.

Conversely, as NRMT1 expression is also found reduced in the stroma of breast tumors [36], we tested if loss of NRMT1 from the surrounding mammary gland niche can also promote tumor cell growth. Wild type and *Nrmt1*^−/−^ C57BL/6 mice were injected with Lewis Lung Carcinoma cells (LLC1) in both the right and left inguinal mammary fat pads and tumor size was measured after one week of growth. LLC1 cells were used in these studies as they are one of the rare tumor cell lines compatible for growth in C57BL/6 mice, and we were interested in assaying how loss of NRMT1 in the mammary niche itself affected tumor growth. We saw no significant difference in the size of the LLC1 tumors between the wild type and *Nrmt1*^−/−^ mice (Figure 6C and 6D), indicating non-autonomous loss of NRMT1 from the mammary niche alone is not sufficient to promote tumor cell growth.

### NRMT1 loss increases MCF-7 sensitivity to tamoxifen

**Figure 7:**
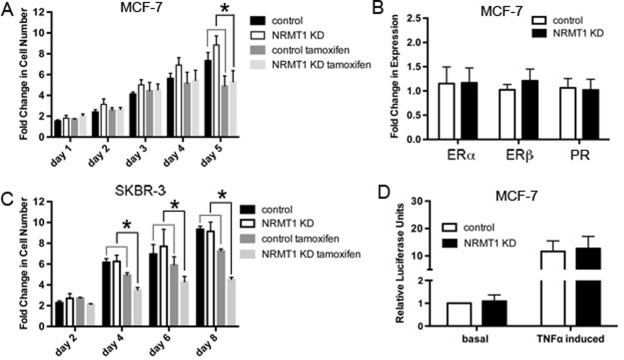
NRMT1 depletion also promotes sensitivity to tamoxifen that is independent of hormone receptor expression levels and NF-κB signaling (**A**) Fold change in cell number of MCF-7 NRMT1 KD and control cells with treatment of 10 μM tamoxifen or vehicle control. Each data point represents the mean ± SEM of three independent experiments. Statistical analysis was by Student's t-test and by comparing the fold change between vehicle treated groups (NRMT1 KD or control) to the corresponding tamoxifen treated groups (NRMT1 KD or control), * denotes p < 0.05. (**B**) RT-PCR analysis of ERα, ERβ, and PR mRNA expression levels normalized to GAPDH in five MCF-7 lines transduced with the NRMT1 KD virus as compared to corresponding control lines. Fold change in expression was calculated by setting control equal to one. (**C**) Fold change in cell number of SKBR-3 NRMT1 KD and control cells with treatment of 10 μM tamoxifen or vehicle control. Each data point represents the mean ± SEM of three independent experiments. Statistical analysis was by Student's t-test and by comparing the fold change between vehicle treated groups (NRMT1 KD or control) to the corresponding tamoxifen treated groups (NRMT1 KD or control), * denotes p < 0.05. (**D**) Luciferase assay demonstrating that neither basal nor TNFα induced NF-κB signaling is increased after NRMT1 knockdown. Each bar represents the mean ± SEM of three independent experiments.

Though tamoxifen is most commonly known for its role as an estrogen receptor antagonist, it can also produce reactive oxygen species (ROS) and DNA breaks [37, 38]. To test if NRMT1 loss subsequently renders MCF-7 cells more sensitive to tamoxifen treatment, lentivirally transduced MCF-7 cells were treated with 10 μM tamoxifen and assayed for viability over five days. As MCF-7 cells treated with NRMT1 KD virus exhibit higher basal growth, we could not directly compare cell viability between control and NRMT1 KD cells treated with tamoxifen. Instead we compared the change in viability for each cell line between untreated and tamoxifen treated. We found that loss of NRMT1 renders MCF-7 cells more sensitive to tamoxifen (Figure 7A), as the difference between untreated and tamoxifen treated NRMT1 KD cells was significantly higher than the difference between untreated and tamoxifen treated control cells. Similar results were seen when treating cells with the tamoxifen metabolite 4-hydroxytamoxifen (Supplementary Figure 1).

To determine how NRMT1 depletion in MCF-7 cells leads to increased tamoxifen sensitivity, we measured the mRNA expression levels of ERα, ERβ, and PR after transduction with the NRMT1 KD or control lentivirus. An increase in endocrine sensitivity is usually accompanied by an increase in the expression of one or more of these three receptors [39]. However, expression levels of all three receptors were unaffected by NRMT1 knockdown (Figure 7B). We then tested whether there was a decrease in NF-κB signaling in NRMT1 knockdown cells, as there is frequently a correlation between increased endocrine sensitivity and inhibition of NF-κB signaling [40], and the oncoprotein NEMO, an essential regulatory subunit of the inhibitor of kB kinase (IKK complex), is a predicted NRMT1 target [3]. Control and NRMT1 knockdown MCF-7 cells were transfected with an NF-κB luciferase reporter and basal luciferase expression was measured, as well as, luciferase levels after induction with TNFα. No difference was seen in NF-κB driven luciferase expression either at the basal level or after TNFα induction (Figure 7D). Lastly, to further demonstrate the increased tamoxifen sensitivity was independent of altered endocrine signaling, we treated control or NRMT1 KD SKBR-3 cells (overexpress HER2 but do not express ER or PR) with 10 μM tamoxifen, as described above, and again saw a significant increase in tamoxifen sensitivity in the NRMT1 KD cells (Figure 7C). As these cells are ER- and PR-, this indicates tamoxifen is inhibiting cell growth through a mechanism alternative from its role as an estrogen receptor antagonist, and we propose this mechanism is increased DNA damage.

## DISCUSSION

It had previously been shown that N-terminal methylation of DNA damage-binding protein 2 (DDB2) is necessary for its recruitment to foci of UV-induced DNA damage and promotes efficient repair of cyclobutane pyrimidine dimers [10]. This supports our initial observation that N-terminal methylation promotes DNA/protein interactions [8]. Here we show that loss of N-terminal methylation promotes sensitivity to two treatments that produce DNA double strand breaks, etoposide and γ-irradiation. Two NRMT1 targets, BRCA1 associated protein 1 (BAP1) and poly-ADP-ribosylase 3 (PARP3), are important in double strand break repair [28-30]. Retinoblastoma protein (Rb) is also a target of NRMT1 [1], though it remains to be seen if N-terminal methylation of Rb regulates is role in the DNA damage response. We propose a model where NRMT1 loss promotes a general decrease in its targets bound to DNA. In regards to DNA repair, this prevents accumulation of important NER and double stranded break repair proteins at sites of damage and results in inefficient resolution of lesions and enhanced sensitivity to DNA damaging agents.

As stated above, the role of N-terminal methylation in DNA repair is not surprising given the accumulating evidence for the involvement of other types of protein methylation. Post-translational modification is a rapid and reversible way to regulate protein function in response to stimuli or damage. A great deal of work has been done describing the role of protein phosphorylation in DNA repair, especially the role of the signaling kinases ATM (ataxia telangiectasia mutated) and ATR (ataxia telangiectasia and Rad3-related protein) in double-stranded break repair [41]. It is now being discovered that there is a significant amount of crosstalk between phosphorylation and other post-translational modifications at sites of DNA damage, including methylation, acetylation, ubiquitination, and SUMOylation [42]. As phosphorylation can be induced and removed in a relatively short timeframe in response to damage [43], it is possible the other modifying enzymes are recruited by phosphoproteins and used to provide a longer-lived, yet still reversible, response. For N-terminal methylation to be used in such a manner, an N-terminal demethylase or protease capable of cleaving α-N methylated amino acids would be required. We predict such an enzyme exists, and it will be interesting to see if it is evolutionarily related to the histone demethylases or a distinct type of demethylase specific for N-terminal methylation. As N-terminal acetyltransferases have been implicated as oncoproteins [44], and we have recently shown that N-terminal methylation and acetylation are not mutually exclusive [3], it will also be interesting to see if the interplay between these two N-terminal post-translational modifications helps dictate cellular fate in response to oncogenic signaling.

We also observed that NRMT1 loss in breast cancer cells increases proliferation, invasive potential, anchorage-independent growth, and xenograft tumor size. NRMT1 loss alone cannot alter the normal growth of MCF10A cells, but it exacerbates the malignant phenotypes of already oncogenic breast cancer cell lines both *in vitro* and *in vivo.* These findings correspond with the levels of NRMT1 protein seen in the various cell lines. Non-oncogenic MCF10A cells have the highest NRMT1 expression, which then decreases in the cancer cell lines with increasing oncogenicity.

Accumulation of DNA damage is one way that NRMT1 loss could promote oncogenic growth. However, NRMT1 loss differentially affects the different types of breast cancer cell lines. Overexpression of DDB2 has opposing effects in ER+ and ER- breast cancer cells and could be one reason by NRMT1 loss differentially affects ER+ and ER- cell lines [45]. Similarly, patients with ER+ tumors have poorer disease outcomes if they have Rb mutation, where ER- negative patients respond better to chemotherapy and have longer relapse free survival when they have Rb mutation [46]. Loss of methylation of Rb could disrupt its interaction with E2F and promote its interaction with ERα, thereby increasing growth in ER+ tumors [47].

In addition to its role in DNA repair, there are other models for why NRMT1 loss most significantly affected growth of the less oncogenic, ER+ MCF-7 and LCC9 cells. These types of tumors are not yet metastatic and are directing their resources to acquiring a growth advantage. As NRMT1 methylates many proteins involved in cell cycle progression and transcriptional regulation, its loss in combination with other oncogenic mutations, could have a profound impact on these processes. Loss of methylation of the oncoprotein SET could promote its interaction with PP2A, thus activating MAP kinase signaling and cell proliferation [35], and we have shown here that levels of phosphorylated ERK do increase upon loss of NRMT1 (Figure 2D). We are currently investigating how N-terminal methylation affects the binding properties of SET. Given that N-terminal methylation is known to promote DNA/protein interactions and NRMT1 is a nuclear methyltransferase, we predict methylation promotes the nuclear histone chaperone activity of SET [48], while loss of N-terminal methylation promotes its cytoplasmic interaction with PP2A.

Alternatively, NRMT1 depletion most significantly affects the invasion and anchorage independent growth of the more oncogenic, ER- SKBR3 and MDA-MB-231 cells. These types of tumors already have a considerable increase in basal growth rates and are directing their resources toward acquiring the ability to metastasize and colonize to secondary locations. Upregulation of the NRMT1 substrate MYL9 correlates with increased invasive potential of MDA-MB-231 cells [49], and we hypothesize that loss of NRMT1 likewise enhances the ability of MYL9 to promote migration by promoting its cytoplasmic localization. In addition to MYL9, there are numerous other myosin light chain proteins that are N-terminally methylated [1], and the increased invasive potential of cells depleted of NRMT1 may result from a cumulative gain in function of these different myosins.

Generation of genomic instability is another hallmark of many breast cancers [50] and may be an additional driving force for the increased oncogenicity seen with lowered NRMT1 expression. We reported a mutant of RCC1 (the guanine nucleotide exchange factor for the small GTPase Ran) that cannot be N-terminally methylated, has decreased binding affinity for DNA, and no longer co-localizes with chromatin [8]. This mislocalization disrupts the Ran-GTP gradient during mitosis and results in multipolar spindles [1, 8] and the formation of viable aneuploid cells [8]. Misregulation of the NRMT1 substrate CENP-A, a prognostic marker for relapse in ER+ breast cancer [51], also leads to multipolar spindle formation [52], and we predict its impaired DNA binding ability after loss of N-terminal methylation [53] may also contribute to the phenotypes seen with NRMT1 loss.

In addition to blocking ER signaling, tamoxifen also produces ROS and DNA breaks [38, 54]. Our data show that NRMT1 loss promotes sensitivity to tamoxifen, without affecting ERα, ERβ, or PR transcript levels or NF-κB signaling. We also show that SKBR-3 cells, which are ER and PR negative, have increased sensitivity to tamoxifen upon NRMT1 KD. We hypothesize that the increased sensitivity of the NRMT1 KD cells to tamoxifen may be due to an additive effect between increased ROS production and a decreased capacity for DNA repair. Taken together, these data indicate that low NRMT1 expression has the potential to serve as a biomarker for patients more likely to respond to a diverse array of DNA damaging chemotherapeutics. As NRMT1 depletion increases sensitivity to tamoxifen treatment without altering ER/PR expression levels, our data also indicate that ER antagonism is not the only mode of tamoxifen action to be exploited, and that combinatorial treatment with other agents that inhibit DNA repair, such as PARP inhibitors, might be a viable option for preventing acquired resistance. Lastly, a potential synergism between tamoxifen treatment and deficiencies in the DNA repair pathway may be one explanation for why patients with inherited BRCA1/2 mutations, who also are deficient in double strand break repair, may benefit from prophylactic tamoxifen treatment independent of ER status [55].

## MATERIALS AND METHODS

### Cell culture

MCF-10A cells (a generous gift from Dr. Lori Millner) were maintained in Dulbecco's Modified Eagle Medium (DMEM): nutrient mixture F-12/phenol red free (Life Technologies, Carlsbad, CA) with 5% horse serum (Life Technologies), 1% penicillin-streptomycin (P/S, CellGro, Manassas, VA), 10 μg/ml of insulin (Sigma, St. Louis, MO), 0.5 μg/ml of hydrocortisone (Sigma), 20 ng/ml of epidermal growth factor (Life Technologies), and 100 ng/ml of cholera toxin (Sigma). MCF-7 and MDA-MB-231 cells were purchased from ATCC. LCC9 cells were kindly provided by Dr. Robert Clarke, Lombardi Cancer Center, Georgetown University [31, 56]. MCF-7, LCC9 and MDA-MB-231 cells were maintained in Improved Minimum Essential Medium (Life Technologies) with 5% fetal bovine serum (FBS, Atlanta Biologicals, Atlanta, GA) and 1% P/S. SKBR-3 cells (generous gift from Dr. Lori Millner) and Lewis Lung Carcinoma cells (LLC1, generous gift from Dr. Yong Li) were maintained in DMEM with 10% FBS and 1% P/S.

### Lentivirus production

GFP-tagged lentivirus expressing an shRNAmir against human NRMT1 or control lentivirus (expressing an shRNAmir against mouse NRMT1) [1] were made by co-transfecting human embryonic kidney cells (HEK293) with 50 μg pGIPZ containing the appropriate shRNAmir, 37.5 μg psPAX2 packaging vector, and 15 μg pMD2.G envelope plasmid using calcium phosphate transfection. Transduction of cells was through addition of the lentivirus to media when passaging using a MOI of 5. After 48 hours, the media was changed and 2 μg/ml of puromycin (Sigma) was added to select for cells infected with virus. Experiments were performed five days post-transduction. Knockdown of NRMT1 was confirmed by western blot and real time PCR. For NRMT1 overexpression, NRMT1 cDNA was cloned into the pWPI lentiviral vector (Addgene, Cambridge, MA). Virus was produced as above, cells were transduced at an MOI of 5, and experiments were performed five days post-transduction.

### Western blots

Primary antibodies used: 1:2000 dilution rabbit anti-NRMT1 [1], 1:5000 dilution mouse anti-tubulin (NeoMarkers, Fremont, CA), and 1:3000 dilution rabbit anti-GAPDH (Trevigen, Gaithersburg, MD). For phospho-ERK immunoblots, cells were starved for 24 hours in serum free media with 1% bovine serum albumin. The next day, the media was changed to that containing 5% FBS and cells were lysed after 15 minutes. Cell lysis buffer included the phosphatase inhibitors sodium fluoride (50 mM), β-glycerophosphate (10 mM), and sodium orthovanadate (0.2 mM, all Sigma). 1:2000 dilution mouse anti-phospho-ERK1/2 (Cell Signaling, Danvers, MA) and 1:1000 dilution rabbit anti-ERK (generous gift from Dr. Alan Cheng) were used as primary antibodies.

### Real time PCR analysis

RNA isolation was performed by cell lysis in TRIzol (Life Technologies). Samples were then mixed with chloroform to extract RNA, the RNA was pelleted using isopropanol, and then washed with ethanol. cDNA was synthesized using the SuperScript First-Strand Synthesis System (Life Technologies). Quantitative RT-PCR was performed with SYBR green PCR Master Mix and the CFX96 Touch^TM^ Real-Time PCR Detection System and Sequence Detection Software (BioRad, Hercules, CA). Primer sequences (Integrated DNA Technologies, Coralville, IA) were as follows; NRMT1 forward 5′ TCTTCCCCCAGGTAGCTCT 3′ and reverse 5′ TGCAGAGGTTTTTAAGGGAAG 3′; ERα forward 5′ TTACTGACCAACCTGGCAGA 3′ and reverse 5′ ATCATGGAGGGTCAAATCCA 3′ [57]; ERβ forward 5′ GCTCCTGTCCCACGTCAG 3′ and reverse 5′ TGGGCATTCAGCATCTCC 3′; PR forward 5′ CGCGCTCTACCCTGCACTC 3′ and reverse 5′ TGAATCCGGCCTCAGGTAGTT 3′ [58] and GAPDH forward 5′ ACAGCCTCAAGATCATCAGCAA 3′ and reverse 5′ CCATCACGCCACAGTTTCC 3′. All real-time PCR assays included analysis of melting curves and agarose gel electrophoresis to confirm the presence of single PCR reaction products.

### Cell growth assays

Cell growth was assayed using the CellTiter 96 AQ_ueous_ One Solution Cell Proliferation Assay (Promega, Madison, WI). Briefly, 1K or 2K cells were plated in triplicate in a 96-well plate and cell number was quantified by addition of 20 μl of AQ_ueous_ One Solution and measurement of absorbance at 490 nm. Relative fold increase was calculated by dividing by absorbance measurements at day zero. For etoposide experiments, MCF-7 cells or LCC9 cells were transduced and 10K cells were plated in triplicate in a 96-well plate. The next day cells were treated with the indicated concentration of etoposide (Sigma) and cell number assayed. For γ-irradiation treatments, MCF-7 cells were transduced, irradiated at 12 or 20 Gy, and 10K cells were plated in triplicate in a 96-well plate. Cell number, 24 and 48 hours post treatment, was measured. For endocrine resistance, MCF-7 or SKBR-3 cells were transduced and the cells were then starved for 48 hours in phenol red-free Improved Minimum Essential Medium with 5% charcoal stripped serum and 1% P/S. Cells were then treated with 10 μM tamoxifen (Enzo Life Sciences, Farmingdale, NY) or methanol as a vehicle control. In the supplemental experiment, MCF-7 cells were treated with 100 nM 4-hydroxytamoxifen (Sigma, St. Louis, MO) or DMSO as a vehicle control. Cell number was measured over a period of 5-8 days.

### Cell cytotoxicity assays

Lactate dehydrogenase (LDH) release was measured using the LDH Cytotoxicity Assay Kit according to the manufacturer's instructions (Cayman Chemical, Ann Arbor, MI). Briefly, conditioned media from 10K cells (grown in IMEM with 1% fetal bovine serum) was incubated with diaphorase/NAD+ mixed with INT (2-(4-iodophenyl)-3-(4-nitrophenyl)-5-phenyl-2H-tetrazolium chloride) and lactic acid for 30 minutes at room temperature. Absorbance was measured at 490 nm. LDH release was calculated using a standard curve.

### Immunofluorescence

MCF-7 cells were transduced with control and NRMT1 knockdown virus as described above. Cells were treated with vehicle control or 240 μM etoposide. At the indicated time points cells were fixed in 4% paraformaldehyde, permeabilized in 0.5% Triton X-100, and blocked in 3% bovine serum albumin. γH2AX staining was performed with 1:250 dilution mouse anti-γH2AX (Novus Biologicals, Littleton, CO) followed by Alexa-Fluro 594-conjugated goat anti-mouse secondary at 1:1000 (Invitrogen). NRMT1 staining was performed with 1:200 dilution rabbit anti-NRMT1 [1] followed by Alexa-Fluro 594 conjugated goat anti-rabbit secondary at 1:1000 (Invitrogen). The cells were counterstained with Hoechst (AnaSpec, Flemont, CA). Cells were visualized using an EVOS FL microscope (Life Technologies). Cells with foci were counted, and the number of foci per cell calculated.

### Migration assay

Cell migration was measured using a scrape motility assay as previously described [59]. Briefly, 24 hours prior to each assay, cells were plated at 100K to 400K in triplicate in a 12-well plate to form a confluent monolayer. Cell layers were scraped using a 1 mL pipette tip (4 scrapes per well). Each scrape was photographed immediately and at the indicated time points (4X, EVOS FL Cell Imaging System, Life Technologies). Scrape widths were measured at the middle and ends of each scrape using Image J software (NIH) and averaged. Distance moved was calculated by subtracting scrape widths at the indicated time points from the initial scrape width.

### Colony formation assay

The ability of cells to form anchorage-independent colonies was measured by plating single cell suspensions in soft agarose. First, a base layer of 0.5% agar (Amresco, Solon, OH) in culture media was added in triplicate to 24-well plates. Cell suspensions were agitated to yield single cells. Cells were plated on top of the base agar at 1.25K in 0.35% agarose (Amresco) in culture media. Cell colonies were fed 1-2 times a week by adding 0.25 ml of culture media. Colonies were allowed to grow for 4 weeks (LCC9, SKBR-3, MDA-MB-231) or 5 weeks (MCF-7). The number of colonies formed was quantified microscopically by counting colonies that were greater than 50 μm in diameter and that were GFP positive (10X, EVOS FL).

### Invasion assay

Cell invasion was quantified using the Trevigen invasion assay (Trevigen, Gaithersburg, MD). Briefly, cells were starved for 24 hours in culture media with 0.1% bovine serum albumin (BSA, VWR, Radnor, VA). After 24 hours, 50K cells in culture media with 0.1% BSA were plated on basement membrane extract in triplicate in the upper chamber of transwell plates. The bottom chamber contained culture media plus 1% FBS as a chemoattractant. After 48 hours, non-invading cells were washed off of the transwell and invading cells were dissociated and stained using a cell dissociation solution/Calcein-AM mixture. The number of invading cells in the dissociation solution was quantified by fluorescence measurements at 485 nm excitation and 520 nm emission.

### Xenograft experiments

The experimental protocol was approved by the University of Louisville School of Medicine Institutional Animal Care and Use Committee. Four-week old female Nu/J mice were obtained from Jackson Laboratories (Bar Harbor, ME). MCF-7 cells were transduced and 3 × 10^6^ NRMT1 knockdown and control MCF-7 cells in 10% Matrigel (BD Biosciences, San Jose, CA) were injected into the mammary fat pads of the NU/J mice. Control cells were implanted into fad pad #9 (right) and experimental cells into fat pad #4 (left). Each mouse was also implanted subcutaneously with a 0.36 mg biodegradable estradiol (E_2_) pellet (Innovative Research of America, Sarasota, FL). NRMT1 knockout mice (*Nrmt1*^−/−^) were generated by the University of Cincinnati Transgenic Mouse Facility and bred to homozygosity at the University of Louisville. C57BL/6J wild type mice were obtained from Jackson Laboratories. 3 × 10^6^ Lewis Lung Carcinoma cells in 10% Matrigel were injected into the mammary fat pads #9 and #4, and tumors were collected one week post-injection. At this time, tumors were excised, photographed, and weighed.

### Luciferase assays

MCF-7 cells were transduced with control and NRMT1 knockdown virus as described above. After puromycin selection, FuGENE HD (Roche) was used to transiently transfect the cells with luc2P/NF-κB-RE/Hygro, which contains five copies of a NF-κB response element, and pGL4-hRluc-TK (*Renilla*, Promega). 24 hours post-transfection, cell lysates were made and the luciferase to *Renilla* signal measured using the Dual-Reporter Luciferase Assay System (Promega) and a VICTOR^3^ multilabel plate reader (Perkin Elmer). The above experiments were also repeated after TNFα (Life Technologies) treatment (10 ng/ml for 6 hours, 24 hours post-transfection).

### Statistical analysis

All statistical analysis was performed using GraphPad Prism Software (La Jolla, CA). The specific statistical test used is noted in the respective figure caption. Results are shown as mean ± standard error unless otherwise noted.

## SUPPLEMENTARY MATERIAL AND FIGURE


