# Supplementary material for: Loss of the N-terminal methyltransferase NRMT1 increases sensitivity to DNA damage and promotes mammary oncogenesis
Source: Oncotarget. 2015 Mar 26;6(14):12248–63. doi: 10.18632/oncotarget.3653 (PMC4494936; doi:10.18632/oncotarget.3653)
Supplement: Supplementary file 1 [file oncotarget-06-12248-s001.pdf]

Loss of the N-terminal methyltransferase NRMT1 increases sensitivity to DNA damage and promotes mammary oncogenesis

Supplementary Material

Supplementary Figure 1

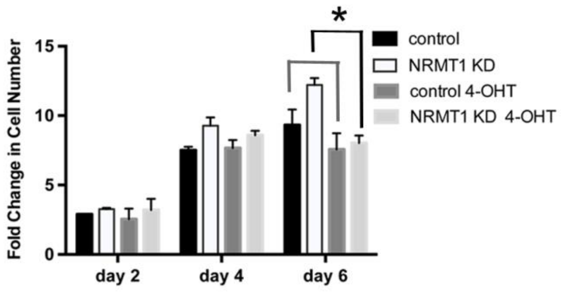

Supplementary Figure 1. Relative fold increase in cell viability of MCF-7 NRMT1 knockdown and control cells with treatment of 100 nM 4-OHT or vehicle control. Each data point represents the mean +/- SEM of three independent experiments. Statistical analysis was by Students' t-test and by comparing the fold change between vehicle treated groups (NRMT1 knockdown and control) to tamoxifen treated groups (NRMT knockdown and control), * denotes $p<0.05$.
